# FTO in Bone Diseases: Functions, Mechanisms and Therapeutic Potential

**DOI:** 10.3390/biom16071035

**Published:** 2026-07-15

**Authors:** Haochuan You, Yixiang Zhao, Xiuyuan Wang, Haotian He, Dacheng Zhao, Yayi Xia

**Affiliations:** 1 Department of Orthopedics, Lanzhou University Second Hospital, Lanzhou 730030, China; youhch2024@lzu.edu.cn (H.Y.); zhyixiang2024@lzu.edu.cn (Y.Z.); wxiuyuan2024@lzu.edu.cn (X.W.); heht2023@lzu.edu.cn (H.H.); 2 Orthopedic Clinical Medical Research Center and Intelligent Orthopedic Industry Technology Center of Gansu Province, Lanzhou 730030, China; 3 The Second School of Clinical Medical, Lanzhou University, Lanzhou 730030, China; 4 Key Laboratory of Dunhuang Medicine and Translation, Ministry of Education, Lanzhou 730030, China

**Keywords:** FTO, m6A demethylation, RNA epitranscriptome, bone metabolism, osteogenic–adipogenic balance, osteoporosis, osteoarthritis, bone-targeted delivery

## Abstract

Skeletal homeostasis relies on coordinated interactions among osteoblasts, osteoclasts, osteocytes, chondrocytes, and bone marrow stromal cells. Disruption of this balance contributes to the development of osteoporosis, osteoarthritis, impaired skeletal repair, and bone malignancies. Fat mass and obesity-associated protein (FTO), an RNA demethylase that removes N6-methyladenosine (m6A) and related RNA modifications, has emerged as a key context-dependent regulator of skeletal biology. Rather than acting uniformly as either a pro-osteogenic or disease-promoting factor, FTO exerts diverse effects that depend on the cell type, disease stage, target transcript, reader-protein context, and mode of therapeutic modulation. This narrative review summarizes current evidence on the role of FTO in osteoblast differentiation, osteoclast activity, bone marrow mesenchymal stem cell (BMSC) lineage commitment, cartilage homeostasis, osteosarcoma, multiple myeloma, and bone-related metastasis. We highlight areas of consensus, unresolved controversies, the strength of the available evidence, and major translational challenges. Collectively, FTO represents a promising therapeutic target in skeletal diseases; however, the current evidence remains largely preclinical and should be interpreted with caution until its efficacy and safety are validated in clinical settings.

## 1. Introduction

FTO (fat mass and obesity-associated protein) was originally characterized as a genetic determinant of body mass, with common variants robustly associated with BMI and obesity risk across diverse populations [1]. Over the past decade, however, an expanding body of work has revealed that FTO functions extend far beyond systemic energy balance, encompassing critical roles in skeletal biology that are only now coming into focus. Bone remodeling—the lifelong, spatially coordinated cycle of osteoblast-mediated formation and osteoclast-driven resorption—is exquisitely sensitive to disruption; its dysregulation constitutes the core pathology of osteoporosis, osteonecrosis, congenital scoliosis, and other debilitating skeletal disorders [2,3]. Central to this process is the lineage commitment of bone marrow mesenchymal stem cells (BMSCs), whose binary choice between osteogenic and adipogenic differentiation determines the balance between bone formation and marrow adiposity. Emerging evidence positions FTO, a principal N6-methyladenosine (m6A) eraser, as a key regulator of this fate decision through its ability to modulate m6A modification on lineage-specifying transcripts [4,5].

Epitranscriptomic regulation, particularly RNA m6A methylation, has emerged as a fundamental layer of gene expression control in skeletal biology. As the founding member of the m6A demethylase family, FTO catalyzes the oxidative removal of m6A marks from mRNA, thereby influencing transcript stability, splicing, and translational efficiency in a spatially and temporally resolved manner [6].

A defining and mechanistically intriguing feature of FTO in bone biology is its profound context-dependent functional duality. Rather than functioning as a simple unidirectional switch, FTO exerts opposing effects depending on the microenvironment. For example, in healthy mesenchymal stem cells undergoing osteogenesis, FTO promotes bone formation by suppressing adipogenic pathways. Conversely, within an osteoporotic microenvironment, altered upstream signaling co-opts FTO to drive adipo-genesis at the expense of bone formation [2,4]. This apparent paradox underscores that the biological output of FTO is not solely determined by its enzymatic activity, but rather by the specific cell type, the developmental stage, the availability of specific target transcripts (e.g., Runx2 versus PPARγ), and the repertoire of downstream m6A “reader” proteins present in the cellular milieu.

In the skeletal system, the substrate repertoire of FTO spans a remarkably broad range of transcripts encoding master transcription factors and signaling molecules that govern osteogenesis, osteoclastogenesis, and dentinogenesis. This review systematically unpacks these context-dependent roles. For instance, FTO directly binds and stabilizes Runx2 (runt-related transcription factor 2) mRNA by protecting it from YTHDF2-mediated decay, thereby promoting cementoblast differentiation and dentin formation [7,8]. FTO additionally regulates the osteogenic differentiation of adipose-derived stem cells (ADSCs) and BMSCs through m6A modulation of FOXO1 and MMP1 transcripts [3,9]. In the osteoclast lineage, FTO upregulation potentiates RANKL-induced osteoclast formation via NF-κB signaling, while FTO depletion suppresses this process [10]. Collectively, these observations position FTO as a central node in the skeletal gene regulatory network whose influence extends across multiple bone-resident cell types.

The advent of high-throughput m6A sequencing and CRISPR-based functional genomics has catalyzed rapid progress in delineating the FTO-regulated molecular network in bone. m6A-RIP-seq and functional studies have identified PPARG, Runx2, and FOXO1 among the key downstream effectors of FTO in osteogenic differentiation [2,5,9]. Concurrently, FTO itself is subject to multilayered regulation by upstream factors including GDF11, PKCβ, and the deubiquitinase USP20 [4,11,12]. In pathological contexts such as inflammatory bowel disease (IBD)-induced bone loss, SUMOylation of FTO modulates BMSC differentiation [13].

While therapeutic targeting of FTO is an area of intense investigation, its clinical readiness for bone diseases remains highly experimental. Moreover, the FTO inhibitor FB23-2 has been shown to suppress osteogenic differentiation of midpalatal suture mesenchymal stem cells during rapid maxillary expansion [14], and small-molecule FTO inhibitors such as CS1 (Bisantrene) have been evaluated in clinical trials for acute myeloid leukemia, highlighting the translational potential of targeting this enzyme [15,16]. It should be noted, however, that Bisantrene is an anthracene derivative originally developed as a DNA-intercalating chemotherapeutic that was subsequently identified as a potent FTO inhibitor [17]. Its FTO-inhibitory activity was discovered retrospectively through structure-based screening. This review discusses current knowledge of FTO in bone metabolism and disease, critically evaluates therapeutic strategies while acknowledging current translational limitations, and identifies key unresolved questions that should guide future investigation.

Scope and literature evaluation: This article is a narrative review. To improve transparency, the searched databases, search period, core search terms, and inclusion logic are stated as follows. We searched [PubMed/Web of Science/Scopus/Google Scholar; please verify] up to [DATE] using combinations of the terms FTO, m6A, bone, osteoblast, osteoclast, BMSC, osteoporosis, osteoarthritis, osteosarcoma, multiple myeloma, and bone metastasis. We prioritized primary mechanistic studies, in vivo validation, disease-relevant models, and recent reviews that clarified field-level controversies.

## 2. Molecular Architecture and Biochemical Functions of FTO

### 2.1. Structural Features and Catalytic Mechanism

**Figure 1:**
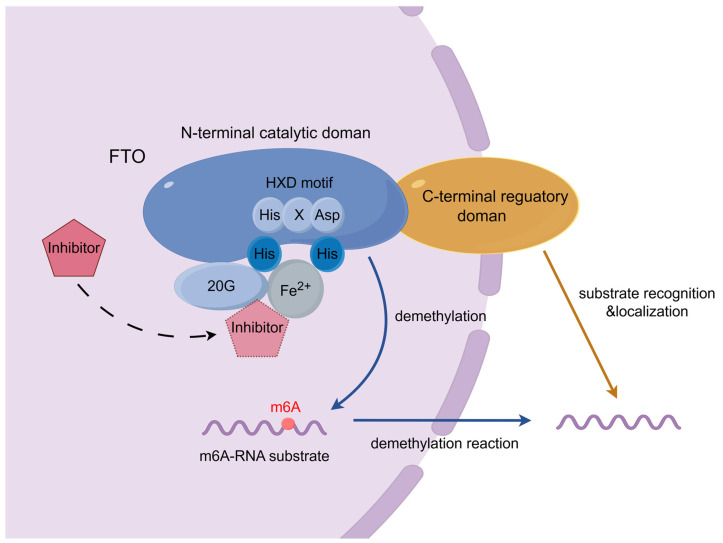
Domain architecture and catalytic mechanism of FTO. FTO comprises an N-terminal catalytic domain (blue) and a C-terminal regulatory domain (orange). The catalytic domain harbors the conserved His-X-Asp (HXD) motif that coordinates the Fe^2+^ cofactor, together with the 2-oxoglutarate (2OG) co-substrate, to execute oxidative demethylation of N^6^-methyladenosine (m6A) on substrate RNA. The C-terminal domain mediates substrate recognition and subcellular localization. Small-molecule inhibitors (red) compete for the catalytic pocket, blocking demethylase activity. m6A marks are dynamically installed by methyltransferases (writers), removed by demethylases (erasers), and interpreted by m6A-binding proteins (readers). Note: The black dashed arrow indicates the binding of the inhibitor to FTO.

FTO belongs to the AlkB family of Fe(II)/2-oxoglutarate (2OG)-dependent dioxygenases and catalyzes the oxidative demethylation of N6-methyladenosine (m6A) in RNA [18]. The N-terminal catalytic domain houses the enzymatically essential His-X-Asp (HXD) motif and two conserved histidine residues that coordinate the Fe^2+^ cofactor; together with 2OG as a co-substrate, these elements enable the oxidative removal of methyl groups from methylated nucleic acid substrates [19,20] (Figure 1). Crystallographic studies have established that the FTO catalytic core accommodates multiple substrates—including 3-methylthymine, 3-methyluracil, and N6-methyladenosine—providing a structural basis for its broad activity across the RNA epitranscriptome [19].

The C-terminal domain of FTO, while catalytically dispensable, plays essential roles in substrate recognition and subcellular localization. Structural and biochemical analyses indicate that this domain contributes to selective RNA substrate binding and may influence the intracellular distribution of FTO, thereby modulating its biological output [16]. Co-crystal structures of FTO in complex with small-molecule inhibitors have revealed that certain compounds occupy both the 2OG co-substrate pocket and the substrate-binding cleft simultaneously, achieving substantially enhanced inhibitory potency [19,20,21] (Figure 1). These structural insights not only deepen our mechanistic understanding of FTO catalysis but also provide a rational foundation for structure-based drug design targeting FTO in obesity, osteoporosis, and cancer [15,18].

### 2.2. Regulation of RNA Methylation by FTO

FTO demethylates RNA with pronounced substrate specificity and sequence context dependence. It preferentially removes m6A from nuclear RNA enriched in the modification, with a marked predilection for the 3′ untranslated region (3′UTR) and sequences flanking stop codons [22,23]. Beyond m6A, the FTO substrate spectrum extends to N6,2′-O-dimethyladenosine (m6Am) in the mRNA 5′ cap and N1-methyladenosine (m1A) [6]. Notably, FTO exhibits higher affinity for m6Am than m6A, and its removal of the 5′ cap m6Am renders transcripts susceptible to DCP2-mediated decapping [24]. Despite this broader substrate range, FTO’s m6A demethylation activity remains the predominant focus in bone research, as m6A is the most abundant internal mRNA modification governing transcript metabolism [25]. FTO activity is also influenced by the RNA structural context: G-quadruplex formation potently inhibits the paralogous demethylase ALKBH5 yet exerts minimal effects on FTO, indicating that FTO can function effectively within complex RNA structural environments [26].

FTO expression and activity are not constitutive but are dynamically regulated by multiple signaling pathways. Metformin, for example, downregulates the methyltransferase METTL3 while upregulating FTO, thereby reducing global m6A levels and impacting lipid accumulation and cell proliferation [27]. Nitric oxide (NO) directly inhibits FTO demethylase activity by chelating the catalytic iron center [28]. In pathological states, including traumatic brain injury and myocardial infarction, FTO expression is markedly downregulated with corresponding alterations in the m6A landscape [29,30]. At the post-transcriptional level, miR-124 suppresses FTO transcription by inhibiting C/EBPα [31], while the zinc finger protein ZBTB48 physically interacts with FTO and mediates its recruitment to specific target RNAs [32]. This multilayered regulatory architecture enables FTO to precisely tune RNA methylation in response to the cellular state and external stimuli.

### 2.3. Integration with the Broader Epigenetic Landscape

FTO does not operate in isolation but functions within a coordinated epigenetic network that governs bone gene expression at multiple regulatory tiers. The dynamic equilibrium between FTO-mediated m6A erasure and METTL3/METTL14-mediated m6A deposition—“writers” and “erasers”—jointly determines the m6A status and hence the expression levels of osteogenic regulators (Runx2 and Bmp2) and osteoclastogenic factors (NFATc1) [10,14]. FTO transcription is itself subject to regulation by histone-modifying enzymes (HDACs) and DNA methyltransferases (DNMTs). At the post-translational level, PKCβ phosphorylates FTO to protect it from ubiquitin-proteasomal degradation [11]. This hierarchical cascade—from DNA methylation and histone modification to RNA methylation—ensures context-appropriate expression of bone metabolic genes under physiological and pathological conditions.

**Figure 2:**
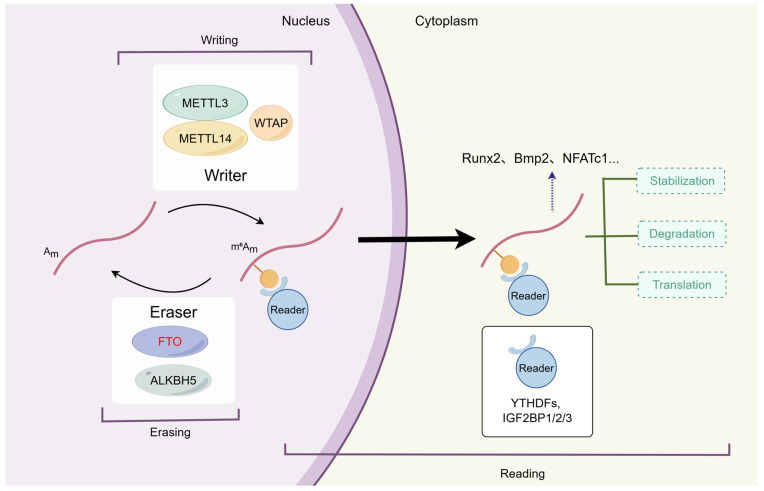
Mechanism of m6A modification. The m6A marks are dynamically installed by methyltransferases (writers), removed by demethylases (erasers), and interpreted by m6A-binding proteins (readers). The m6A-marked mRNA is exported to the cytoplasm, where it is recognized by YTHDF or IGF2BP family proteins (reader) to modulate mRNA stability, degradation, or translational efficiency. In bone metabolism, FTO stabilizes key transcription factor mRNAs (e.g., Runx2, Bmp2, and NFATc1) through m6A demethylation, thereby regulating osteoblast differentiation, osteoclast activation, and BMSC fate determination, serving as a central epitranscriptomic node for bone homeostasis.

Single-cell transcriptomic technologies have begun to reveal pronounced cell-type-specific FTO expression patterns within bone tissue. In osteoblasts, osteoclasts, BMSCs, and osteocytes, FTO levels and functional outputs differ substantially—not merely in expression magnitude but also in the repertoire of m6A reader proteins (YTHDF1-3 and YTHDC1) with which FTO functionally interacts, determining whether demethylated transcripts are routed toward translation or decay [6,10]. The FTO–methyltransferase–reader triad thus constitutes a highly refined “writer-eraser-reader” code that specifies the spatiotemporal expression of bone metabolic genes, offering new opportunities for cell-type-specific therapeutic intervention in skeletal disease (Figure 2).

Beyond the canonical writer–eraser–reader framework, recent structural and functional comparisons between FTO and its paralog ALKBH5 have revealed important distinctions with implications for skeletal biology. Both enzymes, demethylate m6A and ALKBH5, preferentially target transcripts within G-quadruplex-free regions, whereas FTO is largely unaffected by the RNA secondary structure [26]. In the context of glucocorticoid-induced osteonecrosis, ALKBH5—rather than FTO—was identified as the primary m6A eraser stabilizing CX3CL1 mRNA to govern macrophage M1/M2 polarization and preserve bone integrity, suggesting functional compartmentalization between the two demethylases in bone [33]. Furthermore, the balance between METTL3-mediated m6A deposition and FTO-mediated erasure is dynamically regulated during specific stages of osteogenesis. METTL3 regulates alternative splicing of Vegfa in BMSCs to promote osteogenic differentiation [34], while FTO concurrently demethylates a distinct set of lineage-specifying transcripts. This writer–eraser co-regulation, operating on partially overlapping but non-identical transcript pools, establishes a combinatorial m6A code that fine-tunes the spatiotemporal expression of bone-forming genes [33,34].

## 3. FTO Regulation of Bone Metabolism

FTO regulates osteoblast differentiation through m6A demethylation of mRNAs encoding master osteogenic transcription factors, notably Runx2 and Osterix (Sp7). Importantly, while several studies report that FTO-mediated demethylation stabilizes Runx2 mRNA and promotes osteogenesis [2,35], Wang et al. [5] demonstrated the opposite: FTO demethylation of Runx2 mRNA inhibited osteogenic differentiation in BMSCs. This discrepancy may reflect cell-type-specific differences in reader protein expression (e.g., YTHDF1 vs. YTHDF2 dominance) or differential demethylation of distinct m6A sites within Runx2 mRNA [36]. A dedicated discussion of this controversy follows below. During osteogenic differentiation of BMSCs, FTO expression rises significantly, and FTO knockdown suppresses alkaline phosphatase (ALP) activity and mineralized nodule formation [2]. The osteogenic activity of FTO intersects with multiple signaling cascades. FTO modulates the maturation of pri-miR-487a in an m6A-dependent manner, thereby influencing WNT5A-mediated osteogenesis, which uncovers a previously unrecognized mechanism through which epitranscriptomic regulation interfaces with Wnt signaling [37]. Additionally, FTO stabilizes PDIA3 mRNA through demethylation, establishing a PDIA3/FTO/USP20 positive-feedback loop that sustains osteoblast differentiation [12]. FTO also safeguards osteoblast integrity by tempering the Hspa1a-NF-κB axis, protecting cells from genotoxic damage; FTO-deficient osteoblasts exhibit heightened sensitivity to DNA-damaging agents and increased apoptosis [38]. In vivo, conditional deletion of FTO in osteoblasts exacerbates bone loss in mice [38]. Moreover, FTO was shown to promote osteogenic differentiation of human BMSCs through direct demethylation of TGFB2 mRNA by stabilizing this transcript and enhancing TGF-β signaling—a mechanism that operates in parallel with but independently of the Runx2 and PPARγ pathways [39]. Thus, FTO coordinates osteoblast differentiation and function through direct demethylation of lineage-specifying transcription factors, signal pathway modulation, and maintenance of genomic integrity.

FTO exerts complex, context-dependent control over osteoclast differentiation and bone-resorbing activity. These conflicting findings can largely be explained by the divergent experimental models employed. In the ovariectomy (OVX)-induced osteoporosis model, FTO is significantly upregulated in bone marrow-derived monocytes (BMMs) and co-localizes with tartrate-resistant acid phosphatase (TRAP) in femoral tissue [10]. FTO overexpression enhances RANKL-stimulated formation of TRAP-positive multinucleated cells and F-actin rings in RAW264.7 cells, accompanied by upregulation of the master osteoclastogenic transcription factors NFATc1 and c-FOS [10]. Conversely, FTO knockdown or pharmacological inhibition with FB23-2 suppresses osteoclast formation and bone resorption [40]. At the molecular level, FTO reduces m6A modification on NFATc1 and c-Fos mRNAs by stabilizing these transcripts and promoting their translation [10]. When FTO activity is diminished, elevated m6A levels on osteoclastogenic transcripts enhance recruitment of the m6A reader YTHDF2, which directs these mRNAs toward degradation [40]. FTO additionally activates NF-κB signaling by promoting p65 phosphorylation and nuclear translocation, thereby enhancing NF-κB occupancy at the NFATc1 promoter [10]. Importantly, FTO regulation of osteoclasts exhibits tissue and disease specificity. In an ovariectomized mouse model, FTO promoted osteoclast differentiation and bone resorption by activating NF-κB signaling [10]. In stark contrast, other disease models reveal an opposing role for FTO. In ankylosing spondylitis (AS), elevated FTO in mesenchymal stem cells suppresses osteoclastogenesis via the lncRNA NORAD/miR-4284 axis [41]. Furthermore, under pro-osteogenic mechanical stimulation via low-intensity pulsed ultrasound (LIPUS), FTO is upregulated to reduce m6A levels on osteoclast-related RNAs, thereby inhibiting osteoclast differentiation [42].These divergent observations underscore that the net effect of FTO on osteoclast biology is highly contingent on the disease context and cellular microenvironment.

The binary fate choice of BMSCs between osteogenic and adipogenic lineages is a critical determinant of bone mass, and FTO functions as a pivotal molecular switch in this decision. A direct comparison of these processes illustrates FTO’s contextual duality. High FTO expression favors osteogenesis, whereas low expression tilts the balance toward adipogenesis [2]. However, in the highly specific microenvironment of osteoporotic and aged individuals, this dynamic shifts. In osteoporotic and aged individuals, FTO is significantly downregulated in the bone marrow, a decline mechanistically linked to increased BMSC adipogenesis and progressive bone loss [4]. Yet, locally within specific progenitor niches exposed to an osteoporotic milieu, elevated GDF11 expression upregulates FTO via C/EBPα. Here, FTO directly binds and demethylates PPARγ mRNA—the master transcriptional driver of adipogenesis. FTO stabilizes PPARγ mRNA through demethylation, actively promoting adipocyte differentiation while suppressing ALPL and osteopontin (OPN) expression [2,4]. This demonstrates how varying upstream signals (e.g., GDF11) dictate whether FTO drives osteogenesis via Runx2 or adipogenesis via PPARγ. This comparison suggests that the key question is not whether FTO is intrinsically pro-osteogenic or pro-adipogenic but rather which target transcript and reader pathway dominate in a specific BMSC state. Beyond adipogenesis, FTO promotes chondrogenic differentiation of BMSCs by targeting SMAD3 mRNA for demethylation [43]. The functional versatility of FTO in BMSC biology establishes it as a central rheostat whose dysregulation underlies multiple skeletal pathologies. Table 1 summarizes the main downstream targets and functional outcomes of FTO across bone-related cell types.

## 4. FTO in Osteoporosis

### 4.1. FTO Genetic Variants and Bone Mineral Density

Large-scale population genetic studies have identified FTO as a susceptibility locus for osteoporosis. A candidate-gene association analysis in a Chinese Han population found that six SNPs within intron 8 of FTO were significantly associated with hip bone mineral density (BMD), with per-risk-allele effect sizes ranging from 0.015 to 0.025 [52]. A UK Biobank cohort study employing Mendelian randomization and conditional false discovery rate analysis identified 63 pleiotropic loci—including CCDC170, ESR1, and FTO—jointly associated with BMD and breast cancer risk, suggesting shared genetic architecture [53]. Concurrently, integrative bioinformatic analyses combined with experimental validation have identified FTO among a panel of m6A-related diagnostic biomarkers capable of discriminating low-BMD phenotypes in postmenopausal women, reinforcing its clinical potential as a biomarker [54]. These data position FTO as a bona fide genetic determinant of bone density.

FTO expression is altered in osteoporotic bone. In the OVX-induced osteoporosis model, changes in FTO expression coincide with alterations in global m6A methylation levels [10]. In a rat rapid maxillary expansion (RME) model, FTO and osteocalcin (OCN) are co-upregulated in the expanded maxilla, consistent with a role in bone remodeling [14]. Together, these findings establish a close link between FTO expression dynamics and bone metabolic status.

### 4.2. Mechanistic Contributions of FTO to Osteoporosis Pathogenesis

**Figure 3:**
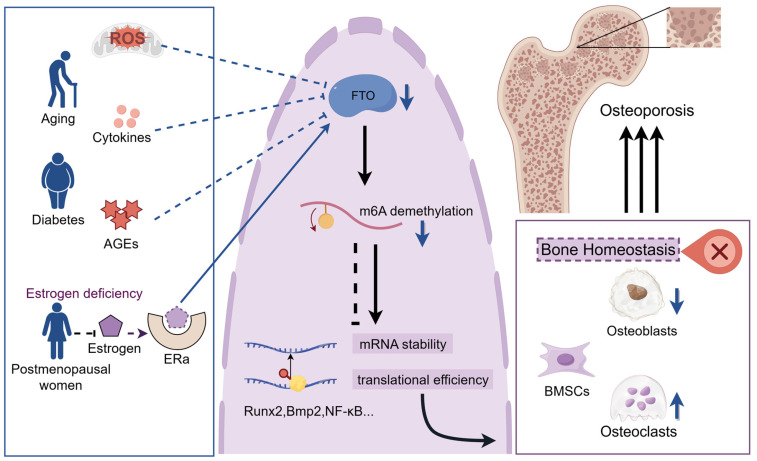
Regulatory role of FTO in the pathogenesis of osteoporosis. In the pathogenesis of osteoporosis, FTO expression and activity are regulated by multiple upstream factors and contribute to bone homeostasis through m6A demethylation-mediated control of target mRNA stability and translation. As illustrated, aging, inflammatory cytokines, reactive oxygen species (ROS), advanced glycation end products (AGEs), and postmenopausal estrogen deficiency suppress FTO expression (↓), whereas estrogen promotes FTO expression through the ERα signaling pathway (↑). As a key m6A demethylase, FTO downregulation increases m6A modification levels on target mRNAs. FTO regulates the expression of key osteogenic- and osteoclastogenic-related genes (e.g., Runx2, Bmp2, and NF-κB), thereby influencing bone cell fate and function. Aberrant FTO expression inhibits osteoblast differentiation and promotes osteoblast apoptosis while enhancing osteoclast differentiation, ultimately disrupting bone metabolic homeostasis. Collectively, these changes impair osteogenesis and enhance osteoclastogenesis, ultimately contributing to osteoporosis. Note: The dashed lines indicate inhibition, the solid lines indicate promotion, and the cross marks indicate dysregulation.

The osteoporotic microenvironment, characterized by enhanced oxidative stress and increased pro-inflammatory cytokine production, disrupts the coupling equilibrium between osteoblasts and osteoclasts [55,56]. Accumulating evidence indicates that FTO may contribute to the regulation of this pathological process (Figure 3). Osteoblast-specific deletion of FTO resulted in age-related bone loss. Mechanistically, FTO regulated the expression of Hspa1a and other DNA repair genes through m6A demethylation. FTO deficiency increased osteoblast susceptibility to oxidative stress-induced DNA damage and apoptosis, accompanied by dysregulation of NF-κB signaling. Under metabolic stress conditions, these effects were further exacerbated [38]. Moreover, in the context of diabetes-associated bone disease, advanced glycation end products (AGEs) suppress FTO expression in BMSCs, leading to m6A hypermethylation and stabilization of sclerostin (SOST) mRNA, a potent inhibitor of Wnt signaling and bone formation [44]. This AGE-FTO-SOST axis provides a mechanistic link between metabolic dysregulation and impaired bone quality in diabetic patients. Consensus clustering analysis of osteoporotic patients identified FTO among 13 differentially expressed m6A regulators, with FTO emerging as a candidate diagnostic gene for low-BMD phenotypes in postmenopausal women [57]. These data implicate inflammatory suppression of FTO in the pathogenesis of metabolic bone loss.

Loss of FTO function elevates m6A modification on mRNAs encoding osteogenic factors, reducing their translational efficiency and compromising osteoblast survival. During RME, FTO is upregulated in the midpalatal suture region where it drives osteogenic differentiation of suture-derived mesenchymal stem cells (SuSCs); FTO knockdown markedly reduces the expression of Runx2, Bmp2, and Col1a1 [14]. This indicates that FTO-mediated demethylation is required to maintain the expression program of bone-forming genes.

In estrogen-deficiency osteoporosis, estrogen positively regulates FTO expression through ERα signaling. Epidemiological data demonstrate that the FTO rs9930506 GG genotype confers a nearly two-fold increase in hip fracture risk in postmenopausal women [58], and specific FTO haplotypes (H1/H9) are associated with lower BMD and an elevated fracture risk [59]. In OVX mice, FTO is upregulated in BMMs and co-localizes with TRAP [10]. Notably, FTO exhibits cell-type-specific expression changes in the osteoporotic microenvironment. Within the BMSC compartment, FTO downregulation may relieve its tonic suppression of osteoclastogenesis [41]; meanwhile, in RANKL-primed osteoclast precursors, FTO is markedly upregulated and drives osteoclast formation through NF-κB-dependent NFATc1 activation [10]. This duality likely reflects cell-type-specific and disease-stage-dependent regulation and warrants further dissection using single-cell-resolution approaches.

### 4.3. Therapeutic Targeting of FTO in Osteoporosis

Pharmacological restoration of FTO function represents a conceptually attractive strategy for correcting the osteogenic–adipogenic imbalance in osteoporosis. FTO knockdown reduces osteoclast formation and bone resorption via NF-κB pathway suppression, increasing trabecular bone volume and BMD in OVX mice [10]. Conversely, in the RME model, the FTO inhibitor FB23-2 suppresses SuSC osteogenic differentiation [14], highlighting that precise modulation is essential for therapeutic benefit. Nanocarrier-based gene and protein delivery systems offer an alternative approach to restore FTO function locally. AAV9-mediated FTO delivery reduces post-ischemic m6A hypermethylation and attenuates brain injury in a stroke model [60]; conceptually, analogous strategies could be deployed in bone. However, FTO is broadly expressed across metabolically active tissues—promoting adipogenesis in adipose [61], lipid accumulation in the liver [62], and modulating cognition and mood-relevant circuits in the brain [63,64]—and acts as an oncogenic driver in multiple cancers [65,66,67]. Systemic FTO activation thus carries a substantial risk of adverse metabolic, neurological, and oncogenic effects. These opposing outcomes indicate that systemic FTO activation or inhibition is unlikely to be appropriate for osteoporosis without targeted delivery, cell-specific control, and careful monitoring of bone formation, resorption, and extra-skeletal toxicity.

## 5. FTO in Osteoarthritis

### 5.1. Regulation of Chondrocyte Metabolism

**Figure 4:**
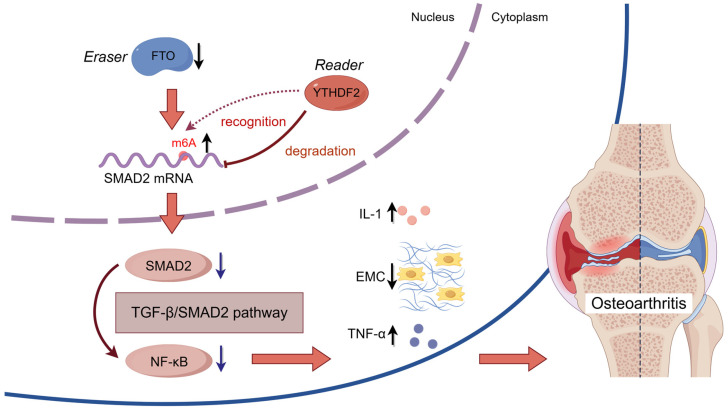
FTO regulates SMAD2/TGF-β signaling through a m6A-YTHDF2-dependent mechanism in osteoarthritis progression. In osteoarthritis (OA) chondrocytes, FTO expression is significantly downregulated (↓), resulting in diminished demethylase activity as an m6A “eraser”. Reduced FTO activity leads to elevated m6A modification levels on SMAD2 mRNA. The m6A reader protein YTHDF2 recognizes and binds to hypermethylated SMAD2 mRNA, promoting its degradation. Decreased SMAD2 protein levels weaken TGF-β/SMAD2 signaling pathway activity, which in turn relieves the inhibition of NF-κB (↑) and reduces extracellular matrix (ECM) synthesis (↓) while increasing its degradation. Ultimately, increased production of inflammatory cytokines (IL-1 and TNF-α), together with ECM homeostasis imbalance, drives the progression of osteoarthritis. This figure illustrates the critical protective role of FTO in OA and its molecular mechanism for maintaining cartilage homeostasis through m6A epitranscriptomic regulation.

Chondrocyte metabolic dysregulation is a central driver of osteoarthritis (OA) pathogenesis, and FTO has emerged as a critical guardian of cartilage homeostasis. FTO expression is significantly downregulated in OA cartilage, and this reduction disrupts the balance between extracellular matrix (ECM) synthesis and degradation [45,47]. FTO mediates m6A demethylation of SMAD2 mRNA, enhancing its stability in a YTHDF2-dependent manner; SMAD2 is indispensable for maintaining the chondrocyte phenotype and promoting ECM anabolism [45] (Figure 4). FTO additionally sustains the expression of SOX9, COL2A1, and ACAN—genes central to chondrocyte identity and function—whose m6A hypermethylation in FTO-deficient chondrocytes silences their expression [46].

Concomitant with the decline in anabolic gene expression, FTO downregulation unleashes catabolic programs. In FTO-low chondrocytes, matrix metalloproteinases (MMPs) and ADAMTS enzymes are transcriptionally derepressed, accelerating degradation of collagen and proteoglycans [46]. FTO regulates the m6A-dependent processing of pri-miR-3591, controlling levels of mature miR-3591-5p, which targets PRKAA2 to govern chondrocyte metabolism and survival [47]. Similarly, FTO overexpression mitigates OA inflammation through the miR-515-5p/TLR4/MyD88/NF-κB axis [68]. In rheumatoid arthritis (RA), an autoimmune inflammatory arthritis condition with secondary OA features, aberrantly elevated FTO in fibroblast-like synoviocytes (FLSs) drives synovial inflammation through the FTO-CMPK2 pathway, activating the mtDNA-cGAS-STING axis and secondarily compromising chondrocyte homeostasis [69]. It is also worth noting that increased global m6A methylation and elevated METTL3 expression were demonstrated in RA synovial tissue, correlating with synovitis severity and inflammatory cytokine production [70]. These findings collectively reveal that FTO operates within a multilayered m6A epitranscriptomic network that finely calibrates chondrocyte anabolic and catabolic activities and that its dysregulation is a principal driver of cartilage degeneration [71].

### 5.2. FTO and Inflammatory Signaling in Osteoarthritis

FTO contributes to chondroprotection in OA in part by restraining NF-κB and MAPK inflammatory cascades. OA cartilage and degenerated chondrocytes exhibit globally elevated m6A levels with concomitant FTO downregulation [45]. FTO overexpression in lipopolysaccharide (LPS)-stimulated chondrocytes enhances proliferation, suppresses apoptosis, and preserves ECM integrity [47]. Mechanistically, FTO-mediated stabilization of SMAD2 mRNA activates TGF-β/SMAD2 signaling, which antagonizes NF-κB-driven production of IL-1β and TNF-α [45]. This places the SMAD2-TGF-β-NF-κB axis at the center of the reported chondroprotective mechanism, although the relative contribution of this pathway versus other FTO-regulated transcripts requires model-specific interpretation.

In IL-1β-challenged chondrocytes, FTO overexpression attenuates NLRP3 inflammasome activity. FTO modulates m6A modification on autophagy-related genes (ATG5 and ATG7) and the pro-apoptotic factor BNIP3 in a YTHDF2-dependent manner—stabilizing ATG5/ATG7 mRNAs while promoting BNIP3 degradation [72]. The resulting enhancement of autophagic flux and suppression of apoptosis eliminate damaged organelles and reduce reactive oxygen species (ROS), thereby interrupting NLRP3 inflammasome activation [72]. Moreover, extracellular vesicles derived from FTO-overexpressing BM-MSCs (FTO-EVs) suppress chondrocyte senescence and apoptosis while promoting protective autophagy in both in vitro and in vivo OA models [72].

In vivo genetic evidence firmly establishes the cartilage-protective role of endogenous FTO. Chondrocyte-specific FTO conditional knockout mice (FTO^flox/flox; Col2a1-Cre) subjected to surgically induced OA exhibit exacerbated cartilage erosion, synovitis, proteoglycan loss, and upregulation of MMP13 and pro-inflammatory cytokines compared to controls [45]. In the meniscus, FTO deletion accelerates degeneration and OA progression, while intra-articular injection of AAV-FTO restores autophagic function, improves energy metabolism, and attenuates meniscal degeneration and OA [73]. These data collectively establish FTO as an essential endogenous chondroprotective factor operating through coordinated suppression of inflammatory signaling and activation of autophagic and metabolic homeostasis pathways.

### 5.3. Therapeutic Implications for Osteoarthritis

FTO-based gene therapy strategies have demonstrated preclinical promise for cartilage repair. AAV-mediated FTO overexpression in meniscal cells restores autophagy and attenuates early OA in mouse models [73]. Natural compounds including resveratrol and curcumin upregulate FTO expression, and flavonoids such as quercetin physically interact with FTO [74,75], suggesting that FTO is a molecular mediator of their chondroprotective effects. BM-MSC-derived FTO-overexpressing extracellular vesicles (FTO-EVs) suppress chondrocyte senescence and apoptosis through METTL3/YTHDF2-mediated m6A modulation [72]. However, FTO-specific RNA activation approaches (e.g., small activating RNAs, saRNAs) have yet to be developed for OA, and intra-articular delivery efficiency remains a significant translational bottleneck. Future efforts must focus on optimizing delivery platforms—including lipid nanoparticles and engineered exosomes—to achieve sustained, cartilage-targeted FTO upregulation.

## 6. FTO in Bone Malignancies

### 6.1. FTO Expression and Function in Osteosarcoma

FTO is significantly overexpressed in osteosarcoma (OS) and correlates with aggressive tumor behavior and poor patient prognoses. Database-driven analyses have identified FTO among the differentially expressed m6A regulators in OS metastatic samples, where its expression level independently predicts overall survival [76,77]. In the MG63 OS cell line, FTO is markedly elevated relative to normal controls [78]. Furthermore, Lv et al. systematically screened 28 m6A regulators and identified FTO as a key m6A demethylase in osteosarcoma. FTO expression was markedly increased in osteosarcoma tissues and cells. Elevated FTO expression was significantly associated with lung metastasis and poor survival outcomes [79].

FTO drives OS proliferation and cell-cycle progression through m6A-dependent stabilization of oncogenic transcripts. In vitro, FTO overexpression promotes osteosarcoma cell proliferation and migration, while FTO depletion reverses these effects. Shan et al. reported that FTO mediates m6A demethylation of KLF3 (Krüppel-like factor 3) mRNA and that KLF3 acts as a tumor suppressor in osteosarcoma [48]. However, the proposed mechanism that FTO decreases KLF3 mRNA stability via a YTHDF2-dependent pathway is difficult to reconcile with the established m6A reader model. YTHDF2 recognizes m6A-marked transcripts and targets them for degradation [80]. Because FTO removes m6A, its activity on KLF3 mRNA would be expected to reduce YTHDF2-mediated decay. This discrepancy from the canonical model means that, although the finding has appeared in a peer-reviewed journal, we advise caution until it is independently replicated. FTO also demethylates P4HB (protein disulfide isomerase) mRNA, activating VEGFA-VEGFR2 signaling to suppress programmed cell death—including apoptosis, ferroptosis, and pyroptosis—while enhancing proliferation, migration, and invasion [49]. TRIM17 ubiquitinates and degrades FTO; the consequent reduction in FTO stabilizes PDK1 mRNA by activating AKT/mTOR signaling to sustain OS malignancy [81]. In metastatic OS, FTO engages epithelial–mesenchymal transition (EMT) programs: FTO knockdown significantly impairs migration and invasion, while Honokiol upregulates FTO and Smad6 to induce autophagy and suppress MG63 cell motility [67]. Furthermore, entacapone, an FDA-approved catechol-O-methyltransferase (COMT) inhibitor, modulated the FTO/DACT1 axis and suppressed osteosarcoma-associated phenotypes in cell and animal models, supporting further preclinical evaluation of its potential as an FTO-targeting agent [79]. In vivo, FTO depletion inhibits pulmonary metastasis of OS xenografts [49].

### 6.2. FTO in Multiple Myeloma

FTO functions as a key oncogenic driver in multiple myeloma (MM). MM patient plasma cells exhibit globally reduced m6A methylation, which is largely attributable to aberrant FTO upregulation [50]. FTO stabilizes HSF1 (heat shock factor 1) mRNA through m6A demethylation, triggering the downstream heat shock protein (HSP) network and promoting MM cell proliferation, migration, and invasion in a YTHDF2-dependent manner [50]. FTO additionally reprograms tumor cell energy metabolism by modulating m6A methylation on metabolic genes including LDHA and PDK1, shifting the balance toward glycolysis to fuel rapid proliferation [51]. Clinically, FTO levels correlate positively with MM bone disease severity: high FTO expression exacerbates osteolytic lesions by disrupting osteoblast and osteoclast function within the bone marrow microenvironment [51].

The central role of FTO in MM pathogenesis positions it as an attractive therapeutic target. Small-molecule inhibitors including FB23-2 and MO-I-500 suppress MM cell proliferation and induce apoptosis in vitro [50]. In xenograft models, FTO inhibition not only curtails tumor growth but also reduces osteolytic bone destruction; combining FTO inhibitors with bortezomib (BTZ) produces synergistic suppression of myeloma bone tumor formation and extramedullary dissemination [50]. Although no FTO-specific inhibitor has yet entered clinical trials for MM, structure-guided drug design has yielded compounds with improved potency and selectivity that warrant further preclinical evaluation [15,16].

### 6.3. FTO in Bone Metastasis

In prostate cancer, FTO loss increases m6A modification and paradoxically enhances cell motility, invasion, and EMT; DDIT4, a downstream target of FTO, is significantly elevated in patients with bone metastases [82]. Within the metastatic microenvironment, tumor-derived FTO may suppress osteoblast activity by altering m6A modification on Runx2 and other osteogenic transcripts while simultaneously promoting osteoclastogenesis via NF-κB activation—establishing a feed-forward “vicious cycle” of bone destruction that accelerates tumor progression [10,14].

## 7. FTO in Other Bone Diseases

### 7.1. FTO in Congenital Skeletal Malformations

Rare missense mutations and copy number variants in FTO have been reported in association with congenital skeletal anomalies including craniosynostosis and brachydactyly; however, the causal molecular mechanisms remain largely undefined. Direct evidence linking FTO mutations to human congenital skeletal malformations is limited, and the current understanding derives primarily from animal models. Zebrafish FTO knockout produces spinal curvature, delayed ossification, and other developmental defects [83], providing suggestive but not definitive parallels to human congenital bone disorders. These phenotypes may arise from an m6A imbalance during early growth plate formation: loss of FTO demethylase activity leads to aberrantly elevated m6A modification on transcripts within key developmental signaling pathways [6,14]. Additionally, given FTO’s central role in energy metabolism and fat deposition [84], FTO mutations could indirectly impair skeletal development by compromising the energy supply and microenvironmental homeostasis required for normal skeletogenesis. Critically, the chain of evidence connecting FTO to human congenital skeletal malformations remains incomplete; future studies must integrate human genetics with refined conditional animal models to determine whether and how FTO mutations disrupt Wnt, BMP, and Hedgehog signaling networks during skeletal morphogenesis.

### 7.2. FTO in Fracture Healing and Bone Regeneration

FTO plays an instrumental role in bone repair. Its expression is dynamically regulated during fracture healing, rising during the early callus formation phase to promote MSC osteogenic commitment. Through m6A demethylation, FTO stabilizes mRNAs encoding osteogenic master regulators including Runx2 and Bmp2, thereby accelerating callus mineralization [14]. In the RME-induced bone remodeling model, FTO knockdown markedly suppresses SuSC osteogenic differentiation [14]. Conversely, FTO deficiency severely impairs fracture repair: FTO knockdown in rats delays femoral fracture healing, with a reduced cartilaginous callus area and retarded bone bridge formation [10]. These dual effects—promoting osteogenesis for callus formation while modulating osteoclast activity for remodeling—position FTO as a central coordinator of the bone repair process.

FTO-based gene-augmentation strategies hold considerable promise for bone regeneration. Local delivery of FTO-overexpressing plasmids or AAV vectors to bone defects enhances new bone formation and accelerates defect closure in critical-size defect models [60]. Additionally, miR-874-3p suppresses human MSC osteogenic differentiation by targeting FTO, suggesting that anti-miR-based derepression of FTO could serve as an alternative therapeutic avenue [85]. Fine-tuning FTO activity—whether through gene therapy, small molecules, or non-coding RNA modulation—may ultimately enable effective treatment of fracture non-union, critical bone defects, and metabolic bone diseases.

### 7.3. FTO in Osteonecrosis

Osteonecrosis, particularly glucocorticoid-induced osteonecrosis of the femoral head (ONFH), represents an important and underexplored area in FTO skeletal biology. While direct studies of FTO in ONFH remain limited, emerging evidence from m6A epitranscriptomic profiling and genetic association studies implicates the m6A machinery, including FTO, in the pathogenesis of ONFH. A candidate-gene association study in a Chinese Han population identified the FTO rs62033406 A>G polymorphism as significantly associated with ONFH risk, providing the first genetic link between FTO variants and osteonecrosis susceptibility [86].

Transcriptome-wide m6A profiling and immune infiltration analysis of ONFH femoral head tissues revealed extensive m6A modification dysregulation, with multiple m6A regulators—including writers, erasers, and readers—differentially expressed between necrotic and healthy bone [86]. Notably, YTHDF3-associated m6A regulation was found to intersect with cuproptosis-related gene expression in steroid-induced ONFH, suggesting that m6A reader proteins mediate programmed cell death pathways beyond canonical apoptosis in the necrotic bone microenvironment [87]. These observations, while not FTO-specific, establish the broader principle that m6A homeostasis is critically disrupted in osteonecrosis.

In related m6A regulatory mechanisms, METTL3 was shown to inhibit BMSC apoptosis and facilitate osteonecrosis repair through an m6A-IGF2BP2-dependent mechanism, enhancing osteogenic differentiation and new bone formation in necrotic lesions [88]. Furthermore, ALKBH5, the paralogous m6A demethylase, governs macrophage polarization and bone integrity in glucocorticoid-induced osteonecrosis through stabilization of CX3CL1 mRNA [33]. Given the structural and functional similarities between FTO and ALKBH5, it is plausible that FTO plays parallel or compensatory roles in ONFH pathogenesis—a hypothesis that awaits experimental testing. The intersection of m6A dysregulation with cuproptosis, ferroptosis, and immune dysregulation in ONFH represents a fertile area for future investigation.

## 8. FTO as a Therapeutic Target: Development and Challenges

Table 2 summarizes reported FTO-modulating agents and gene/cell-based approaches, but the entries should be interpreted by evidence stage and disease context. The therapeutic targeting of FTO in bone disease confronts a bidirectional dilemma. FTO inhibition may be useful in malignant contexts such as osteosarcoma or multiple myeloma, where FTO often supports tumor growth or survival [15,50]. The same inhibitory strategy may be harmful in non-malignant skeletal disorders if it suppresses osteogenesis, cartilage repair, or meniscal homeostasis [14]. Conversely, FTO activation or gene delivery may support selected degenerative or repair settings but could be unsafe in malignancy-prone contexts. Therapeutic proposals should therefore specify the disease context, cell target, modulation direction, delivery route, and evidence level.

Gene- and cell-based strategies offer possible routes to tissue-selective FTO modulation, but most remain at the early stage. CRISPR-dCas9 systems can in principle enable locus-specific FTO expression control [41], and engineered cells or biomaterial scaffolds may improve local delivery [14]. In addition, bone-targeted viral and non-viral delivery platforms, including AAV vectors engineered for enhanced skeletal tropism and lipid nanoparticles functionalized with bone-targeting ligands, are being actively investigated as potential approaches for localized FTO modulation [60,62]. However, AAV immunogenicity, lipid nanoparticle cytotoxicity, limited skeletal tropism, and the need to avoid ectopic FTO activation in cancer-prone tissues [65,66] remain major barriers.

To provide a structured comparison of these therapeutic strategies, Table 3 outlines the mechanisms, disease contexts, developmental stages, and critical limitations of each approach.

The foremost safety concern for clinical translation is FTO pleiotropy. FTO participates in energy metabolism, adipose distribution, neural biology, and tumor behavior. Therefore, systemic FTO modulation risks metabolic perturbation [61,91], fatty liver disease [62], and neuropsychiatric effects [63,64,92,93]. No FTO-based bone therapeutic has entered routine clinical use, and genetic associations between FTO polymorphisms and OA risk remain inconsistent, with some effects mediated through BMI and others appearing obesity-independent [94,95]. The most realistic near-term priorities are bone- or cartilage-specific delivery, inducible expression control, biomarker-guided patient selection, and clear separation of malignant from non-malignant indications [96,97].

## 9. Conclusions and Outlook

### 9.1. Unresolved Controversies

**Figure 5:**
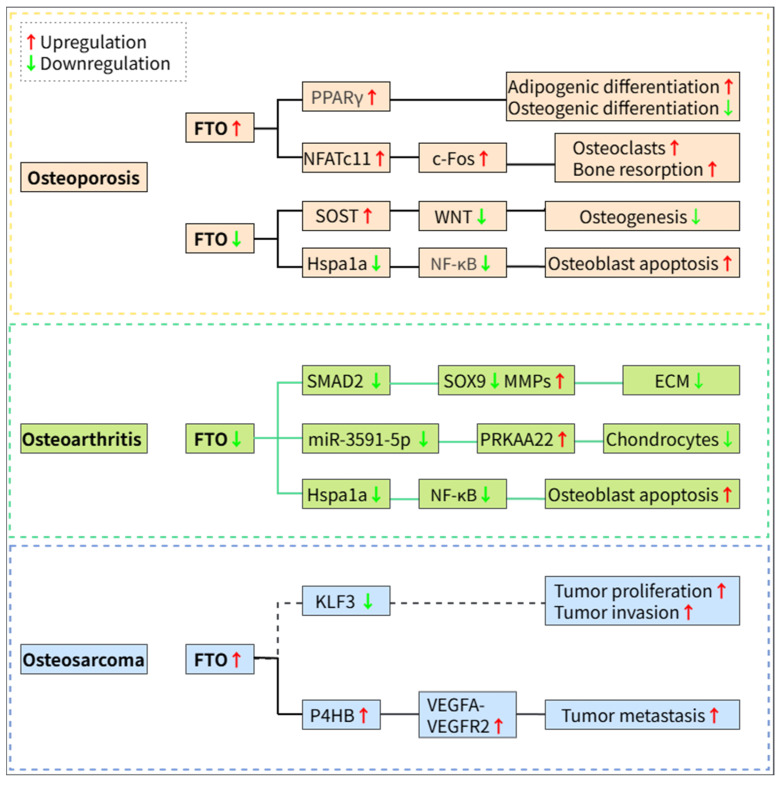
Divergent FTO-mediated regulatory networks across osteoporosis, osteoarthritis, and osteosarcoma. This schematic summarizes reported relationships rather than uniformly established causal pathways. In osteoporosis, FTO has been linked to BMSC adipogenic differentiation through PPARgamma regulation, osteoclast activation through NFATc1/c-Fos-related signaling, and osteoblast stress responses through Hspa1a/NF-κB-associated mechanisms. In osteoarthritis, reduced FTO activity can destabilize SMAD2, weaken TGF-β/SMAD2 signaling, relieve NF-κB activity, and increase matrix-degrading enzymes. In osteosarcoma, FTO-associated m6A regulation has been linked to KLF3- and P4HB/VEGFA-related tumor programs, although the KLF3/YTHDF2 directionality should be interpreted cautiously and verified against the original study. Note: The pathways illustrated in this figure represent relationships reported in individual studies. Solid arrows denote interactions supported by direct experimental evidence (knockdown/overexpression, m6A-RIP-qPCR, and reporter assays) in the indicated cell type and disease context. Dashed or gray arrows indicate relationships that are inferred, context-dependent, or require independent validation. Readers should interpret these networks as working models rather than as definitive, universally applicable signaling maps.

Several fundamental questions remain unresolved in FTO bone biology. First, FTO can promote osteogenesis in some settings while promoting adipogenesis in osteoporotic BMSCs, indicating that the target transcript and the cellular state must be interpreted together [2,4,35]. Second, FTO may enhance osteoclast formation in OVX/RANKL contexts but suppress osteoclastogenesis through MSC-mediated paracrine mechanisms in ankylosing spondylitis models [10,42]. Third, reported FTO expression changes in osteoporosis differ across sampling times, disease stages, and cell populations [10,57,98]. Fourth, many studies assign phenotypes to m6A without fully excluding FTO effects on other substrates such as m6Am or m1A. Resolving these issues will require time-course single-cell profiling, conditional models in defined skeletal lineages, substrate-specific assays, and direct comparisons of m6A reader engagement. Figure 5 provides an overview of the divergent FTO-mediated regulatory networks involved in osteoporosis, osteoarthritis, and osteosarcoma, integrating the relationships reported across different studies.

### 9.2. Outlook

Four priorities should guide the next stage of FTO bone research. First, single-cell and spatial approaches should define where FTO is expressed and which transcripts it controls in each skeletal cell population [99]. Second, multi-omics studies should integrate m6A, m6Am, transcript abundance, translation, and protein output to separate direct FTO substrates from downstream effects [99]. Third, disease-specific delivery systems should be developed for bone, cartilage, marrow, or tumor compartments, with inducible control to reduce systemic risk. Fourth, clinical biomarker studies should test whether FTO expression or m6A signatures predict the disease stage, treatment response, or adverse effects in well-defined cohorts [96,97].

Finally, FTO should be interpreted within the broader writer–eraser–reader network rather than as an isolated therapeutic switch. The expanding literature on m6A modification in skeletal disease indicates that METTL3/METTL14, ALKBH5, YTHDF proteins, IGF2BP proteins, and FTO may act in parallel or opposing directions depending on the cell state [100,101]. Future therapeutic strategies should therefore target validated disease-specific networks, not FTO expression alone.

### 9.3. Conclusions

FTO is best understood as a context-sensitive epitranscriptomic regulator rather than as a uniformly beneficial or harmful skeletal factor. Across osteoblasts, osteoclasts, BMSCs, chondrocytes, and bone tumor cells, its net effect depends on cell identity, disease microenvironment, target transcript, reader-protein context, and whether FTO activity is increased or inhibited. This framework explains why FTO inhibition may be attractive in selected bone malignancies but potentially detrimental in cartilage repair or osteogenic regeneration. A clinically useful FTO strategy will therefore require evidence-based matching of the disease context, modulation direction, and delivery specificity.

## Figures and Tables

**Table 1:** FTO downstream targets and functions across bone-related cell types.

Cell Type	FTO Change	Key Target(s)	Molecular Mechanism	Functions	Ref.
Pre-osteoblasts	↑	Runx2 and Osterix	m6A demethylation → mRNA stabilization	Osteogenic differentiation ↑	[2,35]
Osteoblasts	↓	Hspa1a	m6A ↑ → mRNA destabilization → NF-κB pathway weakened	Apoptosis ↑; bone formation ↓	[38]
BMSCs	↑	PPARγ	m6A demethylation → PPARγ mRNA stabilization	Adipogenesis ↑; osteogenesis ↓	[2,4]
BMSCs	↓	Runx2 and Bmp2 SOST	m6A ↑ → reduced osteogenic gene expression	Osteogenic differentiation ↓	[2,14,44]
Pre-osteoclasts	↑	NFATc1 and c-Fos	m6A demethylation → mRNA stabilization; NF-κB p65 activation	Osteoclastogenesis ↑; bone resorption ↑	[10]
Osteoclasts	↓	Cyclin A2 and CDK2	m6A ↑ → YTHDF2-mediated degradation	Osteoclast formation ↓	[40]
Chondrocytes	↓	SMAD2	m6A ↑ → YTHDF2 degradation → SMAD2 ↓	ECM degradation ↑; SOX9 ↓, COL2A1 ↓	[45,46]
Chondrocytes	↓	pri-miR-3591	m6A-dependent miRNA processing impaired	miR-3591-5p ↓ → PRKAA2 dysregulation	[47]
Osteosarcoma cells	↑	KLF3	m6A demethylation → YTHDF2 degradation	Proliferation ↑; invasion ↑	[48]
Osteosarcoma cells	↑	P4HB	m6A demethylation → P4HB ↑ → VEGFA-VEGFR2↑	Anti-death; metastasis ↑	[49]
Myeloma plasma cells	↑	HSF1	m6A demethylation → HSF1 ↑ → HSPs activation	Proliferation ↑; osteolytic bone disease ↑	[50,51]

Note: The arrow (↑) denotes upregulation/increase; (↓) denotes downregulation/decrease. Reference numbers correspond to the main text bibliography. Evidence-strength note: The table summarizes reported FTO-associated effects across models. Direct in vivo evidence, cell-culture mechanistic evidence, and inferred pathway relationships should be distinguished when interpreting these entries.

**Table 2:** Compendium of reported FTO small-molecule inhibitors and agonists.

Compound	Category	Mechanism of Action	Bone-Related Effects	Ref.
FB23-2	Inhibitor	Competes with 2OG co-substrate; occupies substrate-binding pocket	Suppresses osteoclastogenesis; inhibits SuSC osteogenic differentiation in an RME model	[14,15,40]
Rhein	Inhibitor	Natural anthraquinone; binds FTO catalytic domain	Inhibits FTO demethylase activity in vitro; bone-specific effects remain uncharacterized.	[83]
Meclofenamic acid (MA)	Inhibitor	NSAID; occupies FTO substrate-binding site	Reduces FTO expression in spinal dorsal horns; alleviates bone cancer pain in rat models	[89]
CS1 (Bisantrene)	Inhibitor	Bis-anthracene; spans both 2OG and substrate sites	Anti-tumor activity in AML; FTO inhibition validated in clinical trials	[15,16]
Entacapone	Inhibitor	FDA-approved COMT inhibitor; drug repurposing candidate	Inhibits osteosarcoma progression via the FTO/DACT1 axis	[79,90]
Quercetin	Inhibitor	Natural flavonoid; physical interaction with FTO	Physical interaction with FTO demonstrated in silico; direct bone-related functional data are lacking.	[74,75]
AAV-FTO	Agonist (gene therapy)	Adeno-associated virus-mediated FTO overexpression	Restores autophagy in meniscus; alleviates OA progression in a mouse model	[73]
FTO-EVs	Agonist (cell therapy)	BMSC-derived extracellular vesicles overexpressing FTO	Inhibits chondrocyte senescence and apoptosis; triggers protective autophagy in OA	[72]

Abbreviations: 2OG, 2-oxoglutarate; SuSC, sutural mesenchymal stem cell; RME, rapid maxillary expansion; NSAID, non-steroidal anti-inflammatory drug; AML, acute myeloid leukemia; COMT, catechol-O-methyltransferase; OA, osteoarthritis; AAV, adeno-associated virus; BMSC, bone marrow mesenchymal stem cell; EV, extracellular vesicle. Reference numbers correspond to the main text bibliography.

**Table 3:** Structured comparison of FTO-targeted therapeutic approaches.

Approach	Mechanism of Action	Primary Disease Context	Stage of Development	Major Limitations and Concerns
Small-Molecule Inhibitors (e.g., FB23-2 and Entacapone)	Blocks FTO catalytic activity/substrate binding	Osteosarcoma and Multiple Myeloma	Preclinical (except for drug repurposing)	Systemic toxicity; impairs normal osteogenesis/cartilage repair; lack of bone specificity.
Gene Therapy (AAV-FTO)	Restores FTO expression locally	Osteoarthritis and Osteoporosis	Preclinical (animal models only)	AAV immunogenicity; potential oncogenic risk if expression escapes skeletal targeting.
Cell Therapy (FTO-EVs)	Delivers FTO mRNA/protein via exosomes	Osteoarthritis	Early in vitro/in vivo	Scalability, standardization of the EV cargo, and variable tissue retention.

## Data Availability

The original contributions presented in this study are included in the article. Further inquiries can be directed to the corresponding authors.
